# Perinatal transmission of *Borrelia burgdorferi:* advancing scientific and clinical understanding of Lyme disease in pregnancy

**DOI:** 10.3389/fmed.2026.1794120

**Published:** 2026-04-09

**Authors:** Sue Faber, Charlotte Mao, Elizabeth Darling, Holly Ahern, Catherine Brissette, Tessa D. Gardner, Janis J. Weis, Sven Bergström, Edward B. Breitschwerdt, Carolyn B. Coyne, Brian A. Fallon, Holiday Goodreau, John S. Lambert, Vett Lloyd, Alessandra Luchini, Sarah B. Mulkey, Kate Nagel, Bennett Nemser, Natalie Rudenko, Jinyu Shan, Michal Caspi Tal, Monica E. Embers

**Affiliations:** 1 LymeHope, Burlington, ON, Canada; 2 Bay Area Lyme Foundation, Portola Valley, CA, United States; 3 Department of Obstetrics and Gynecology, McMaster University, Hamilton, ON, Canada; 4 Science Division, State University of New York Adirondack, Queensbury, NY, United States; 5 Department of Biomedical Sciences, University of North Dakota, Grand Forks, ND, United States; 6 Mercy Hospital, Saint Louis, MO, United States; 7 Department of Pathology, Division of Microbiology and Immunology, University of Utah, Salt Lake City, UT, United States; 8 Department of Molecular Biology, Umeå University, Umeå, Sweden; 9 Department of Clinical Sciences, College of Veterinary Medicine, North Carolina State University, Raleigh, NC, United States; 10 Duke University School of Medicine, Durham, NC, United States; 11 Department of Psychiatry, Lyme and Tick-borne Diseases Research Center, Columbia University,New York, NY, United States; 12 LivLyme Foundation, Denver, CO, United States; 13 Rotunda Hospital, Mater Misericordiae University Hospital, University College Dublin, Dublin, Ireland; 14 Department of Biology, Mount Allison University, Sackville, NB, Canada; 15 School of Systems Biology, George Mason University, Manassas, VA, United States; 16 Children’s National Hospital, George Washington University School of Medicine and Health Sciences, Washington, DC, United States; 17 LymeLight Foundation, Burlingame, CA, United States; 18 Steven & Alexandra Cohen Foundation, Stamford, CT, United States; 19 Institute of Parasitology, Biology Center, Czech Academy of Sciences, České Budějovice, Czechia; 20 Phelix Research and Development, London, United Kingdom; 21 Micropathology Ltd, University of Warwick Science Park, Coventry, United Kingdom; 22 Department of Biological Engineering, Massachusetts Institute of Technology, Cambridge, MA, United States; 23 Department of Microbiology and Immunology, Tulane University Health Sciences, New Orleans, LA, United States

**Keywords:** *Borrelia (Borreliella) burgdorferi*, congenital, Lyme disease, pregnancy, perinatal transmission

## Abstract

Perinatal transmission of *Borrelia burgdorferi* sensu lato (Bb), the spirochetal agent of Lyme disease, is an issue of public health importance and research significance. This alternate mode of transmission and the potential risk of adverse pregnancy outcomes were communicated within public health spheres following the first suspected case in 1985. Subsequent studies in reservoir and non-reservoir animal hosts, in addition to case reports of perinatal morbidity and mortality in humans brought further attention to the field. Decades later, however, the incidence and epidemiologic impact of perinatal transmission of Bb, as well as the clinical spectrum and potential long-term health sequelae of gestationally exposed children, remain understudied and poorly defined. In June 2022, a Banbury Conference on Perinatal Transmission of Lyme disease was convened at Cold Spring Harbor Laboratory in New York. This manuscript conveys conference findings and research recommendations to advance scientific and clinical understanding of this important issue.

## Introduction

Lyme disease (LD) is a globally distributed tick-borne infectious disease caused by several species of *Borrelia* spirochetes (1). *B. burgdorferi* sensu lato refers to a group of over 20 related genospecies associated with Lyme borreliosis worldwide, while *B. burgdorferi* sensu stricto is the primary Lyme disease agent in North America. LD is currently the most prevalent vector-borne disease in the United States (US) and Europe, and the number and distribution of new cases continues to rise (1). In the US, close to half a million cases of LD are diagnosed and treated yearly (1). Global seroprevalence is estimated at 14.5% with the highest rates identified in Central Europe, Western Europe and Eastern Asia (2).

**FIGURE 1:**
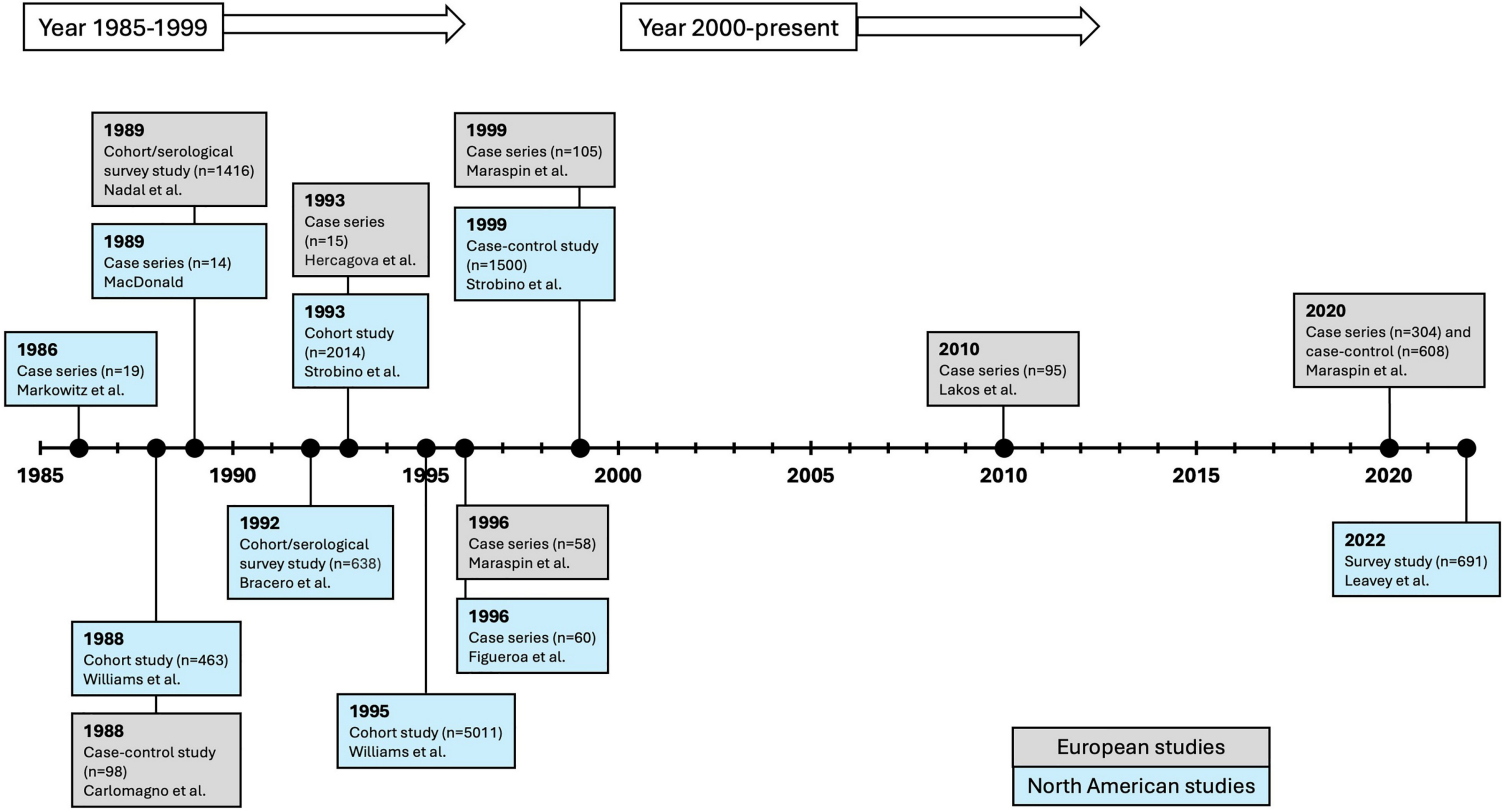
Timeline of Lyme disease and pregnancy epidemiologic/clinical studies published to date. Blue boxes represent studies carried out within North America and gray boxes are studies from Europe. The majority of this research was conducted over a 13-year time span. A striking research void is observed from the year 2000 onward. Individual case reports are not included in this timeline.

In 1981, the tick-borne spirochete now known as *Borrelia burgdorferi* was identified as the cause of LD (3). Shortly thereafter, *Bb* was discovered to be capable of crossing the placental barrier in humans and infecting the fetus (4), followed by additional reports in both humans (5–19) and animals (20–30). Perinatal transmission of *Bb* and the potential risk of fetal/infant morbidity and mortality were conveyed through international public health bulletins (31, 32), expert reviews (3, 33–41), and publications from professional societies (42–44). Human clinical and epidemiologic investigations pertaining to LD and pregnancy were initiated (9, 18, 45–56), but unfortunately, by the year 2000, new research in the field had significantly declined (Figure 1).

Currently, *Bb* is recognized as one of several microbial agents capable of causing *in utero* infections (57), acknowledged by the US Centers for Disease Control (CDC), the US National Institutes of Health and the Public Health Agency of Canada (58). Generally regarded as rare (58), and lacking a clearly defined congenital syndrome (59), the true incidence of congenital *Bb* infection and the associated clinical spectrum of disease remains poorly understood and substantially understudied (58). The percentage of LD cases worldwide that includes pregnant women and women of child-bearing age who may potentially infect their offspring is not known. Perinatal transmission of *Bb* could contribute to the rising global disease burden, with clinical and epidemiologic consequences (60, 61).

In June 2022, the Banbury Center at Cold Spring Harbor Laboratory (New York, United States) hosted a meeting of international Lyme disease experts representing academia, clinical medicine, government agencies, and foundations. Meeting objectives included a review of the existing literature and knowledge on the specific issue of perinatal (vertical) transmission of *Bb*, which we defined as transmission during pregnancy, birth, or the postpartum period. Given the limited body of evidence pertaining to perinatal transmission of LD, and to be as comprehensive as possible, we included conference abstracts and book chapters, as well as peer-reviewed research. Herein, the Banbury conference findings are reviewed and resulting research recommendations are proposed.

## Pathogenesis

The pathogenesis of LD is an important consideration when assessing its impact on pregnant women and their children. *Bb* shares many pathophysiologic similarities with other spirochetes including the species of *Borrelia* which are the agents of relapsing fever and *Treponema pallidum* (Tp), the agent of syphilis (62–64). During hematogenous dissemination in the mother, these pathogenic spirochetes can also cross the placenta, which may result in pregnancy complications, congenital infection and fetal death (33, 62, 64, 65).

*Borrelia burgdorferi* and other *Borrelia* species can cause vascular damage in multiple organs, either directly or indirectly, by triggering host inflammatory responses (66). Tissue damage at the maternal-fetal interface may compromise placental barriers, potentially facilitating pathogen invasion of the placenta and impairing blood and nutrient flow to the fetus. *Borrelia* spirochetes have been shown to readily adhere to, and pass through the endothelial cells that line blood vessels (67) and induce upregulation of endothelial adhesion molecules for immune cell trafficking (68).

Over 30% of neonatal deaths, primarily in low- and middle-income countries, are caused by infectious disease. Pregnancy complications such as low birth weight, preterm delivery, spontaneous abortion and perinatal death are commonly caused by the relapsing fever (RF) species *Borrelia duttonii* in sub-Saharan Africa (69). *B. duttonii* infection during pregnancy results in intrauterine growth restriction as well as placental damage and inflammation (70). Studies comparing the severity of infection of *Bb* and the RF species *B. duttonii* in pregnant and non-pregnant mice have shown that pregnancy has a protective effect on the severity of maternal disease (64, 71). For example, *Bb*-infected pregnant mice had a significant reduction in arthritis severity, likely as a result of progesterone-induced IL-4 production and a shift toward Th2 responses, although fetal outcomes and the risk of vertical transmission were not studied (71). With *B. duttonii*, despite the attenuation of maternal disease, transmission of the spirochete occurred in 73% of fetuses with additional findings of intrauterine growth restriction, impaired fetal circulation, and decreased maternal hemoglobin levels (64). While this RF species does not pose a threat in the U.S., other RF species (*B. hermsii* and *B. turicatae*) transmitted by soft ticks exist in the western half of the U.S. and have been associated with pregnancy complications (72, 73). Of significant importance is the RF species transmitted by the blacklegged tick, *B. miyamotoi*. This species is increasing in prevalence and *Borrelia miyamotoi* disease is a likely cause of maternal and fetal morbidity possibly attributed to Lyme LD (74). This warrants further research, given the known and anticipated emergence of *B. miyamotoi* (75).

Similarities between *Bb* and *Tp* include the possibility of silent spirochetemia within the context of latent gestational infection (14, 76), and histologic observations of Hofbauer cells in placental villi (33, 77). With *Tp* infections, interactions between the bacteria and the endothelium initiate an inflammatory cascade that can lead to perivascular inflammation and induce the lesions associated with congenital syphilis (65). Likewise, *Bb* has outer surface lipoproteins that are known to be potent inducers of host inflammatory responses (78). With both infections, spirochetes have been identified in some fetal tissues with a minimal or no accompanying inflammatory response (4, 7, 11, 12, 55, 77, 79), possibly related to an immature fetal immune system at the time of spirochetal invasion (33, 80). The impact of pregnancy and hormonal shifts on the innate and adaptive immune response to *Bb* remains understudied. Cytokine and auto-antibody responses to the spirochete during human pregnancy have not been characterized. Interleukin-1β is elevated in LD (81) and has been directly associated with pre-term labor in primates (82). A three-fold increase in the probability of stillbirth has also been associated with autoimmunity in the form of antiphospholipid antibodies (83). Importantly, elevation of antiphospholipid antibodies has been reported in patients and mice with LD (84).

## Insights from animal studies

Vertical transmission of *Bb*, reproductive failure, and offspring mortality have been both reported (20–30) (Table 1) and refuted (85–88) in studies of natural and experimental infections of reservoir and non-reservoir animal hosts. Contradictory findings may be explained by variables associated with fetal exposure to pathogen, including the infecting *Borrelia* strain and its associated pathogenicity, route of infection, inoculation dose, stage of gestational infection and placental type (29). Furthermore, detection of fetal infection or immune response may be influenced by the age of offspring at the time of testing, types and numbers of tissues sampled, and sensitivity and specificity of detection methods.

**TABLE 1:** Vertical transmission of *Borrelia burgdorferi* in animal studies.

References	Animal	Infection type	Detection methods*	Vertical transmission findings
Anderson et al. (20)	Mice	N	C, DfM, IFA with mAb	*Bb* cultured from *P. leucopus* mouse fetus and corresponding maternal spleen and kidney.
Burgess (21)	Cows	N	C, DfM, IFA with mAb	*Bb* cultured from colostrum of seronegative cow that aborted and from blood of seronegative newborn calf. *Bb* antibodies found in aborted calf.
Burgess and Windberg (22)	Coyotes	N	C, DfM, IFA with mAb	Spirochetes with typical *Bb* sp. morphology cultured from coyote fetus (kidney) of seronegative female coyote. Unable to obtain pure subculture for strain identification.
Burgess et al. (23)	Horses	N	C, IFA with mAb	*Bb* cultured from kidney of seronegative foal #1, found dead next to placenta. In foal #2, euthanized at 2 days of age, *Bb* was cultured from kidney and brain and identified by IFA in glomeruli.
Ubico-Navas (24)	Swiss white mice	E	C, DfM, FA	*Bb* cultured from 5/10 unborn term pups from one inoculated dam who died during parturition; 3/4 stillborn pups from two inoculated dams, and 77/98 liveborn pups born to inoculated dams, raised by uninoculated dams, and sacrificed 6–7 weeks post maternal inoculation.
Gustafson et al. (26)	Dogs	E	C, DfM, PCR	Eight infected females delivered litters with at least one neonatal or 6-weeks-old pup with *Bb* positive tissues by PCR, including 4 pups from 3 separate litters (a stillborn, and pups who died at 30 min, 20 h and 48 h). *Bb* also cultured from liver of pup who survived to 20 h.
Burgess et al. (25)	Mice	N	C, DfM, PCR	In fetal tissues removed from uteri of 2 pregnant mice, *Bb* cultured from 2/5 fetuses from *M. musculus* and 1/2 fetuses from *P. leucopus*.
Silver et al. (27)	C3H/HeN mice	E	PCR	*Bb* detected by PCR in 1/3 fetuses and 1/2 placentas in mice infected with *Bb* 5 days prior to mating and sacrificed at day 14 gestation.
Altaie et al. (28)	C3H/HeJ mice (splenectomized)	E	C, DfM, PCR	Phase 1: Maternal inoculation early gestation - *Bb* detected by PCR in 4/30 fetuses and 3/30 placentas; mid-gestation - 3/57 fetuses and 4/57 placentas; late gestation - *Bb* not detected in fetus or placenta. Phase 2: *Bb* detected by culture in fetal organs/tissues. 5/49 (10.2%) of infected females transmitted *Bb* to pups either *in utero* or intrapartum. 2/25 (8%) of infected females transmitted *Bb* to their pups on day 1 via their milk.
Leibstein et al. (29)	Cows	N	C, DfM, PCR	Three stillborn calves showed evidence of disseminated *Bb* infection in multiple tissues by PCR. *Bb* also cultured from spleen of one stillborn calf and kidney of second stillborn calf. Two live-born calves were spirochetemic (by PCR). *Bb* detected by PCR in colostrum of 4/12 cows, including 3 of 4 cows spirochetemic at parturition. *Bb* cultured from placentas of 2/10 cows, and uterine fluid collected at parturition in 1/8 cows.
Wan et al. (30)	Mice and rats	N	C, DfM, IFA with mAb	*Bb* cultured from one fetal striped field mouse (*Apodemus agrarius*) and two fetal white-bellied giant rats (*Rattus edwardsi*)

*Bb*, *Borrelia burgdorferi*;

*C, culture; DfM, darkfield microscopy; E, experimental study; FA, fluorescent antibody test; IFA, indirect immunofluorescent assay; N, naturally infected; mAb, monoclonal antibody H5332; PCR, polymerase chain reaction.

Vertical transmission of *Bb* in certain species of mice and rats was reported from field studies of reservoir hosts in the US and China (20, 25, 30). A 20% rate of stillbirth was demonstrated in a naturally-infected first-calf dairy cattle herd with evidence of disseminated *Bb* in stillborn tissue, placenta, uterine fluid, and live-born calf blood samples (29). Researchers studying naturally-infected pregnant mares reported outcomes of abortion, fetal resorption and foal mortality, with *Bb* recovered by culture from two foals who died shortly after birth (23).

In an experimental mouse study, acute infection of mice at day four or five of pregnancy was associated with fetal loss in 12%–14% of gestational sacs, compared to none of controls (27). This was not observed in mice infected 3 weeks prior to pregnancy. Uteri from acutely, but not chronically, infected mice were positive for *Bb* DNA at 14 days gestation (27). *Bb* DNA was detected in one of three pups and one of two placentas from mice infected with *Bb* 5 days prior to mating; however, vertical transmission of the spirochete did not correlate with fetal loss. This study reveals the potential for *Bb*-induced fetal death, but suggests that altered immune responses and heightened inflammation could also be responsible for pathogenesis leading to fetal death (27). A second experimental murine study demonstrated that 10.2% of infected females transmitted *Bb* either *in utero* or intrapartum to a portion of their pups (28). In eight of ten female beagles intradermally inoculated with *Bb* before breeding, *Bb* DNA positive tissues were found in at least one pup in each litter. Intrauterine transmission of *Bb* was identified in four pups who died less than 2 days of age from three separate litters, with *Bb* also cultured from the liver of one pup who survived to 20 h (26).

Transmission of *Bb* through maternal milk is also of potential concern (87). *Bb* has been detected by polymerase chain reaction (PCR) in cow colostrum and milk (29, 89), and cultured from cow colostrum (21). Oral transmission of *Bb* to pups through maternal milk was reported in an experimental mouse model (28), and infection of adult *Peromyscus* mice with an oral solution of *Bb* was also demonstrated experimentally (90). The mammary glands of C3H mice infected by subcutaneous inoculation of flank skin pre-conception were found to be colonized by *Bb* postpartum (88). Furthermore, recent research found significant and lasting *Bb* infections in the reproductive tracts of non-pregnant female mice, leading to various pathological changes, with older reproductively senescent mice showing more severe effects (91).

A mathematical model of the spread of *Bb* in populations of black-legged ticks and their vertebrate hosts found that the efficiency of reservoir (small mammal) host vertical transmission of *Bb* was one factor strongly impacting the rate of increase and eventual prevalence of LD in natural populations (92). This mode of transmission may be a means by which the spirochete is naturally maintained among hosts for ticks within an enzootic cycle (25, 30).

## Women’s reproductive health and obstetrics

The impact of LD on women’s reproductive health has received little study. An early review of the histopathology of LD included a recollection of cases of “decidual necrosis with inflammation in patients with intrauterine infection due to *Bb*” by one of the authors of a book chapter (77). Authors highlighted uterine involvement in LD as a significant factor in pregnancy, with the potential for *Bb* transmission to the fetus (77). A recent analysis of human health records revealed that LD is associated with an increased risk of several gynecological conditions, including menorrhagia, miscarriage, uterine fibroids, and endometriosis (91).

Of confirmed or probable cases of LD in females of child-bearing age reported to the US CDC between 1992 and 2019, 0.6% were identified as pregnant, most often presenting with an erythema migrans (EM) rash, although 70% of records lacked documentation of pregnancy status (93). Differences have been noted in some clinical features of LD in pregnant versus non-pregnant women. For example, European *Borrelia* strains are less likely to manifest a ring-like EM rash or flu-like symptoms in pregnant women (94). Pregnancy-specific complications reported in association with gestational LD include toxemia/pre-eclampsia, (12, 94) vaginal bleeding (12, 55, 94), placental abruption (95), miscarriage (10, 12, 45, 55, 96), and stillbirth (12, 55).

Information regarding *Bb* seroprevalence in pregnancy is limited to older studies using single-tier antibody testing, with data from US or European studies showing maternal or cord blood seroprevalence ranging between 0.4% and 12% in endemic areas (46–50, 52). A study of pregnant women living in an endemic region of Italy found double the *Bb* seroprevalence rate in the group with spontaneous abortions compared to those with normal term pregnancies (12.2% versus 6.1%) (45).

The current standard diagnostic test for LD is two-tier serology which detects antibodies produced in response to infection by *Bb* (1), although some experts emphasize the importance in pregnancy of a clinical diagnosis irrespective of serologic status (37–39, 41, 97). A pregnant woman with positive *Bb* serology may be asymptomatic (98), or, conversely, she may have negative serology despite evidence of present or past LD (8, 13, 44). Negative or equivocal maternal serology has been reported in conjunction with congenital LD in newborns (8, 13, 41), with histological findings of *Bb* in fetal autopsy tissues (8, 12), cellular immune reactivity to *Bb* in newborns/infants (41), and PCR confirmation of *Bb* in placentas from asymptomatic mothers (53). Routine prenatal screening for LD in endemic areas has been suggested by some experts (45, 98), whereas others recommend against it, advising that based on their data, additional study is necessary (47, 49).

Clinically, gestational LD may go undiagnosed in the absence of an EM rash (52) (which may be absent up to 40% of the time) (1), when the mother is asymptomatic or has non-specific symptoms (6, 8, 12–14, 19, 55, 98, 99), or in instances of maternal seronegativity (8, 12, 13, 41). This poses a serious diagnostic dilemma, as the spirochete can be transmitted to the placenta or fetus in the absence of maternal clinical signs and symptoms (14). In a study of asymptomatic pregnant women in a LD endemic area, 5% of placentas harbored *Bb* spirochetes, implying fetal transmission (53). The authors emphasized the importance of long-term follow-up of exposed infants to determine the potential impact of placental spirochetes on child growth and development (53). Furthermore, European *Borrelia* species have been cultured from the blood of seven pregnant women who presented with a solitary EM rash only, indicating that hematogenous spirochetal dissemination can occur in the absence of systemic symptoms (100). Additionally, in a large cross-sectional international self-reported survey of women with and without LD in pregnancy, many respondents with diagnosed or suspected LD did not receive a diagnosis or treatment until many years after delivery, despite being symptomatic before pregnancy (101). In our review of documented cases of confirmed or possible perinatal transmission of *Bb* from the scientific literature (Supplementary Table 1), 30% (19/62) of mothers had no identified history of LD, and in several cases were asymptomatic.

*Borrelia* DNA was detected in breastmilk from two lactating women with an untreated EM rash, although the spirochete was not cultured (102). Breast milk transmission of *Bb* in humans has not been reported but this lack of data cannot exclude the potential for this mode of transmission (103, 104). Some experts suggest discussing the risks and benefits of breastfeeding with the lactating mother, possibly delaying breastfeeding until LD treatment starts or finishes, and closely monitoring the infant for symptoms suggestive of infection (103, 104).

Healthy newborn outcomes have been described in several studies, most commonly in cases of maternal diagnosis and treatment for LD (12, 18, 41, 47, 51, 54, 55, 94, 98, 100, 105). Prompt diagnosis and treatment of LD in pregnancy is indirectly associated with significantly fewer adverse pregnancy or birth outcomes (11% of treated versus 50% of untreated) (106); however, evidence to guide maternal antibiotic treatment for gestational LD is sparse. Current guidelines from the Infectious Diseases Society of America recommend similar treatment for pregnant versus non-pregnant women, cautioning doxycycline safety in pregnancy requires additional study (59). Some European experts currently suggest or use intravenous antibiotics in all stages of gestational LD (94, 105). Unfortunately, *Bb* has been identified in placental (12, 15–18, 96, 107–109) and fetal tissue (11, 96) from mothers who received a single course, or in some cases multiple or prolonged courses of antibiotic therapy for LD prior to, or during pregnancy (16, 17, 96). The possibility of treatment failure and spirochetal persistence (16, 17, 96, 109) underscores the critical importance of identifying the most appropriate type, route and length of therapy to both treat the mother and prevent possible perinatal transmission of the spirochete (110).

## Fetal and postnatal health of offspring

A congenital syndrome with a defined pattern of clinical signs and symptoms associated with perinatal *Bb* exposure has not been determined (59). However, the true clinical and pathological spectrum of congenital LD may only become more evident with further study. A wide range of adverse pediatric outcomes reported with LD in pregnancy include preterm birth (4, 9, 55, 94, 95), hyperbilirubinemia (9, 18, 41, 46, 55, 105), newborn respiratory distress/suspected sepsis (4, 11, 12, 14, 41, 55, 94), cardiac (4–7, 12, 41, 47, 51, 52, 55, 94) and genitourinary malformations (51, 52, 55, 94, 105) dermatologic (6, 9, 19, 41, 98, 105), ophthalmologic (6, 15, 41), and orthopedic/musculoskeletal anomalies (6, 12, 41, 50, 105), hypotonia (41, 105), hydrocephalus (12, 18, 55, 99), neurologic abnormalities (6, 9, 12, 13, 15, 41, 51, 94, 105, 111), and perinatal death (4, 8, 11, 12, 55, 94).

Some early cases documenting perinatal transmission of *Bb* employed less precise methods of silver staining or darkfield microscopy to detect spirochetes in fetal or placental tissues (4, 55, 94). Additional cases were diagnosed by more specific *Bb* detection methodologies including immunohistochemistry/immunofluorescence assays utilizing *Bb*-specific monoclonal antibodies (7, 11, 18), PCR (15, 19, 53, 63, 96, 107–109), and culture (5, 7, 8, 10, 12, 108, 109) in placenta or post-mortem tissues from cases of miscarriage (7, 10, 12, 63, 96), intrauterine fetal death/stillbirth (5, 7, 12, 18), neonatal death (8, 11, 12), and early (8, 11, 12, 19) or later (15, 19, 111) disease manifestations (Table 2 and Supplementary Table 1). Overall, spirochetes have been identified histologically or cultured from fetal/infant heart, brain, meninges, subarachnoid space, bone marrow, kidneys, spleen, adrenal glands, lungs and liver (4, 7, 8, 10–12, 55). Post-mortem histologic examination of ten cases of sudden infant death syndrome revealed spirochetes morphologically compatible with *Bb* in brain tissue of two infants who died at age 4 months (12). Several larger studies reporting adverse outcomes associated with gestational LD notably could not assess potential causality due to limited or no *Borrelia* testing of exposed infants, placentas, products of conception, or autopsy samples (9, 58, 94, 105).

**TABLE 2:** Outcomes in human cases of perinatal transmission of *Borrelia burgdorferi* with laboratory evidence.

Outcome category	Number of cases	References
Miscarriage/abortion	9	MacDonald (5)
		Neubert (10)
		MacDonald (12)
		Hercogová and Vanousová (63)
		Horowitz and Yunker (96)
		Hulínská et al. (108)
IUFD/stillbirth	6	MacDonald (5)
		MacDonald et al. (7)
		MacDonald (12)
		Hercogová et al. (18)
		Maraspin et al. (55)
Death - early neonatal	6	Schlesinger et al. (4)
		Lavoie et al. (8)
		Weber et al. (11)
		MacDonald (12)
		Maraspin et al. (55)
Death - beyond neonatal period	3	MacDonald (12)
		Spector et al. (15)
IUGR/LBW	5	Lampert (6)
		MacDonald (12)
		Gardner (41)
		Lazebnik and Zal’tsman (111)
Malformations/ anomalies - cardiac	8	Schlesinger et al. (4)
		MacDonald (5)
		MacDonald et al. (7)
		MacDonald (12)
Malformations/ anomalies - other	6	MacDonald (12)
		Gardner (41)
		Maraspin et al. (55)
		Önk et al. (99)
Early manifestations (neonatal period < 4 wk.)	15	Schlesinger et al. (4)
		Lampert (6)
		Lavoie et al. (8)
		Weber et al. (11)
		MacDonald (12)
		Dattwyler et al. (13)
		Horst (14)
		Trevisan et al. (19)
		Gardner (41)
Later manifestations	5	Spector et al. (15)
		Trevisan et al. (19)
Later manifestations		Gardner (41)
		Lazebnik and Zal’tsman (111)
Normal perinatal outcome	6	Patmas (17)
		Hercogová et al. (18)
		Figueroa et al. (53)
		Hulínská et al. (108)

IUFD, intrauterine fetal demise; IUGR, intrauterine growth restriction; LBW, low birthweight.

Based on known organ system manifestations of *Bb* infection in adults and children, cardiac and neurologic disease might logically be predicted as possible sequelae of congenital disease (40). Concern for a possible association between maternal LD and an increased risk of fetal cardiac anomalies (38, 41, 112) is raised by data from case reports and case series (4, 7, 12, 94), as well as two population-based prospective studies (50, 52). In the first study, cardiac anomalies occurred at twice the frequency in infants born in high risk versus low risk towns for LD, although the difference was not statistically significant (50). The second investigation found a statistically significant 2.4-fold increased frequency of cardiac malformations in an endemic cohort of infants compared to controls (52). Both studies concluded that congenital malformations as a whole, as well as fetal death and low birth weight, were not associated with maternal or cord blood seropositivity for LD; however, limitations included small sample sizes for each specific teratogenic diagnosis, unknown sensitivity of serologic testing in neonates, lack of placental examination or other direct testing for presence of *Bb*, and lack of long-term follow-up and monitoring of exposed offspring (50, 52, 113). A later retrospective case-control study from a single pediatric cardiology service found no association between pediatric congenital heart defects and maternal LD within three months before or during pregnancy (56).

Clinical signs and symptoms of congenital LD can be delayed, appearing weeks, months or years after birth (19, 41, 111, 114). Reported manifestations of congenital infection beyond the neonatal period are few but include a variety of neurologic manifestations in children, including developmental delay/loss of developmental milestones, persistent small head circumference, lower extremity spasticity, and sensory and motor polyneuropathy with pronounced autonomic dysfunction and psychological and/or emotional lability (41, 111). Ocular pathologic findings similar to those described in congenital syphilis were reported in a 7-years-old boy who died from cerebral complications of congenital LD (15). Dermatologic manifestations were also reported in a baby with a relapsing multiple annular erythema between age 3 months and 3 years with skin biopsy-confirmed *Bb* by PCR testing (19). Sclerotic transverse metaphyseal bands were demonstrated on long bone x-rays of two neonates diagnosed with congenital LD and disappeared after antibiotic treatment (41).

An international survey of women with and without LD in pregnancy revealed that only 3% of pregnancies at risk of perinatal transmission had testing of placenta or fetal/newborn samples after birth or miscarriage, of which 14% were equivocal or positive results (101). Babies born to mothers treated for LD before or during pregnancy had better health outcomes compared to untreated pregnancies. However, in both treated and untreated LD pregnancies, as children aged, substantially greater rates of fevers, rashes, concentration difficulties, and sensory, respiratory, cardiovascular, musculoskeletal, gastrointestinal, orthopedic, and vision issues were reported in comparison to non-LD affected pregnancies (101).

When LD is suspected of contributing to fetal/neonatal infection or mortality, the placenta and/or fetal/infant organs and tissues should be examined for spirochetes utilizing *Borrelia*-specific direct detection methods and for histologic abnormalities (4, 12, 14, 38, 40, 41, 44, 45, 97). A retrospective search for evidence of maternal or infant LD in liveborn or stillborn infants with congenital cardiac anomalies may be warranted (38). While some consensus guidelines do not recommend additional monitoring of the mother or infant beyond standard of care (59), other experts recommend close evaluation of exposed infants, including investigation for cardiac malformations (38, 112), and long-term follow-up for possible later clinical manifestations of disease (41, 101, 104, 114).

Past testing recommendations for newborns with gestational LD exposure included performing both IgM and IgG enzyme immunoassays (EIAs) and immunoblots on paired maternal and cord blood and on infant blood and cerebrospinal fluid (CSF) (41), as well as culture and/or PCR of infant blood and CSF (40, 41). While demonstration of *Bb*-specific IgM antibodies in cord blood or infant serum at birth is indicative of congenital infection (97), serological testing should not be considered a definitive diagnostic tool or an adequate endpoint to rule out fetal infection (12, 36), and reliance on seropositivity alone may result in misdiagnosis (41). A differing pattern of antibodies identified on immunoblot of infant versus maternal serum may indicate *in utero* infection (34); conversely, a matching maternal and neonate immunoblot pattern suggests *Bb* IgG antibodies detected in the newborn are of maternal origin (105). When antibody responses are not detected or provide equivocal results, T-lymphocytes harvested from cord/infant blood may be useful in testing for cell-mediated immunity to *Bb* (34, 41). Urine antigen testing may also have utility in identifying cases of congenital LD (115).

It is unknown whether congenitally infected individuals might demonstrate a different serologic profile when compared to children or adults who were infected via a tick bite. An infant’s immature immune system or other mechanisms (e.g., immunological tolerance) could impact the immune response in cases of *in utero* infection. In two studies of individual children, one diagnosed with congenital neuroborreliosis (111) and the other with dermatologic manifestations of possible congenital LD (19), the subjects had negative serologic tests, but were diagnosed based on PCR positivity of serum (111) and a skin biopsy (19) sample, respectively. Three additional infants with negative serology were diagnosed with congenital LD using testing for cell-mediated immunity to *Bb*, through a lymphocyte proliferative assay (41). *Bb* antibodies were found in the CSF of two symptomatic neonates, one with neurologic dysfunction (13) and the other with septic disease (14). Samples of placenta and/or cord blood were PCR positive in three cases in which the mother had been treated for an EM rash in pregnancy (107).

Current guidelines from US medical societies state that there is a lack of evidence for congenital infection or a congenital Lyme syndrome and, therefore, make no diagnostic or treatment recommendations for perinatally exposed infants (59). Preliminary guidelines for the diagnosis and management of pregnant women with LD and their at-risk infants had previously been authored by the Infectious Diseases Society for Obstetrics and Gynecology (44), an expert from the CDC (97), and an infectious disease pediatrician (41), but are no longer readily available. Updated comprehensive interim guidelines are needed (58). Currently, no international surveillance system exists to track cases of congenital Lyme disease (98). Institution of a stand-alone ICD-11 code for congenital LD has been debated (113, 116).

Overall, a standardized case definition for identifying congenital LD has yet to be delineated (58), although clinical manifestations of early mild, early severe, and late congenital LD have been categorized into a preliminary framework (41). Prospective studies are needed to better understand clinical outcomes for a mother with LD and her baby (36, 41, 53, 58, 101, 113, 117, 118). Increased healthcare practitioner awareness and education regarding gestational LD (58, 93, 117, 118) and the possibility of congenital infection (97), along with the establishment of pregnancy registries in partnership with the obstetrics and gynecology departments of academic teaching hospitals, have been recommended (112).

## Recommendations and conclusions

**FIGURE 2:**
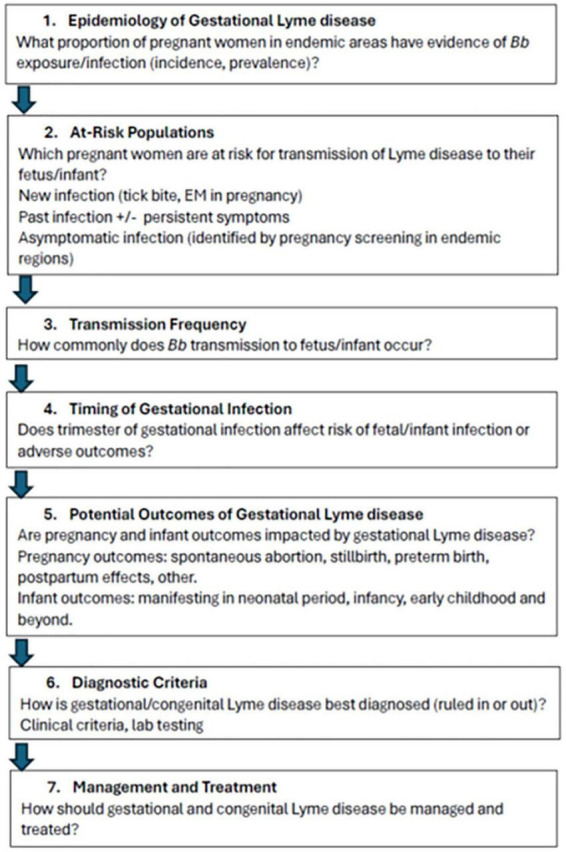
Fundamental research questions addressing perinatal transmission of *Borrelia burgdorferi* in humans.

Perinatal LD is an issue of public health importance and research significance. The totality of existing evidence indicates that *Bb* can cross the placenta and may be associated with adverse pregnancy outcomes including fetal/infant morbidity and mortality, yet many fundamental research questions remain (Figure 2).

**FIGURE 3:**
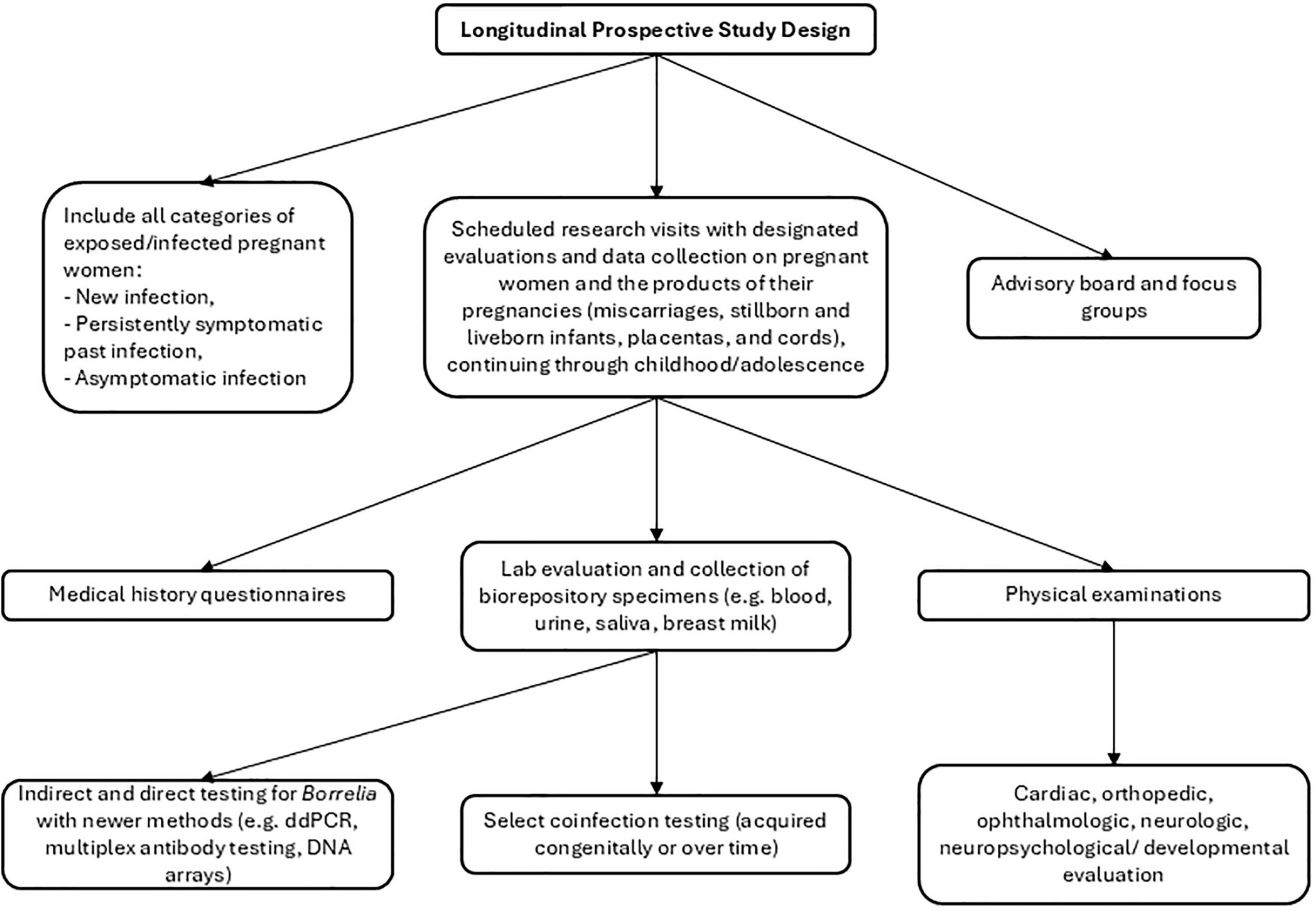
Recommended design for a long-term prospective cohort study of *Borrelia*-infected pregnant women and their infants/children. Specimens should also be banked in a biorepository for additional future analyses as new research questions or better testing methods arise. Community advisory boards and focus groups should include partnerships with parents and other stakeholders.

Our group developed several recommendations to advance the field:

1. *Animal studies (murine, canine, primate) to clarify the pathobiology of LD in pregnancy and congenitally infected offspring*. Evaluation of (a) potential vertical transmission of *Bb* in both acute and late-stage disease; (b) possible transmission through breast milk; (c) efficacy of pregnancy-safe antimicrobial therapy for maternal disease and prevention of fetal transmission; and (d) maternal and neonatal immune responses to infection, and possible immune-mediated mechanisms involved in both early and late presentations of disease acquired perinatally.
2. *Longitudinal prospective studies of pregnant women with LD and long-term follow-up of liveborn infants*. This necessitates the development of standardized case definitions for gestational, fetal, and congenital LD, including clinical and laboratory criteria (119). Cases of fetal death or stillbirth require comprehensive evaluation for potential teratogenic and other adverse effects of *Bb*, including utilization of modern methods for direct detection of *Borrelia* spirochetes in placental and fetal tissues. Biorepository specimen collection, inclusive of maternal and infant blood, CSF, and other specimens (placenta, amniotic fluid, breast milk, urine, saliva and tissue) can be utilized for current or future studies (Figure 3).
3. *Development and validation of existing and new diagnostic testing approaches that enable accurate identification of gestational and congenital LD cases*. These approaches may include evaluation of paired maternal-infant samples using next-generation serological assays, cellular immune testing and/or urine antigen tests. Newer direct testing methods such as droplet digital PCR, DNA hybridization microarrays and phage testing could be useful as well.
4. *Development of clinical guidelines for evaluation and management of pregnant women with LD and their at-risk infants*. Comprehensive guidelines for diagnosis, treatment, and long-term management of pregnant women with LD and newborn infants are urgently needed, similar to those developed for other vector-borne infections such as Zika virus and West Nile virus. These clinical guidelines would be based on a comprehensive synthesis of existing evidence and updated as new evidence emerges.

Since the Banbury meeting, efforts to address research gaps in the field are already underway. The 2022 US Tickborne Disease Working Group (TBDWG) Report to Congress included a section on pregnancy and LD (58). The US Department of Defense Congressionally Directed Medical Research Program (CDMRP) Tick-borne Disease Research Program (TBDRP) included both gestational LD and maternal-to-fetal transmission of *Bb* within its call for research applications (120). Recent studies have examined the impact of gestational LD on obstetrical or infant/child health outcomes (95, 101), as well as research priorities of patients with LD during pregnancy (117). Additionally, a comprehensive proposal for designing prospective studies on gestational LD has been published (119). Investigators at Children’s National Hospital in Washington DC are currently examining the effects of LD on pregnancy and childhood neurodevelopmental outcomes (118).

As the prevalence of LD continues to escalate globally, the identification of accurate diagnostics and effective clinical management for both mother and baby must be prioritized with a coordinated multi-disciplinary, multi-institutional research and policy response. Scientific and clinical advances within the field will lead to new avenues for improved surveillance, health care professional education, and evidence-based diagnostic, prevention, and treatment strategies, providing hope and healing for impacted women and their children.
